# Technology-Mediated Experiences and Social Context: Relevant Needs in Private Vs. Public Interaction and the Importance of Others for Positive Affect

**DOI:** 10.3389/fpsyg.2021.718315

**Published:** 2021-09-01

**Authors:** Pia von Terzi, Stefan Tretter, Alarith Uhde, Marc Hassenzahl, Sarah Diefenbach

**Affiliations:** ^1^Department of Psychology, Ludwig-Maximilians-Universität München, Munich, Germany; ^2^Ubiquitous Design Experience and Interaction, Universität Siegen, Siegen, Germany

**Keywords:** human-computer interaction, psychological needs, need fulfillment, social context, public space, positive affect, user experience, social acceptability

## Abstract

Technologies, such as smartphones or wearables, take a central role in our daily lives. Making their use meaningful and enjoyable requires a better understanding of the prerequisites and underpinnings of positive experiences with such technologies. So far, a focus had been on the users themselves, that is, their individual goals, desires, feelings, and acceptance. However, technology is often used in a social context, observed by others or even used in interaction with others, and thus shapes social dynamics considerably. In the present paper, we start from the notion that meaningful and/or enjoyable experiences (i.e., wellbeing) are a major outcome of technology use. We investigate how these experiences are further shaped by social context, such as potential spectators. More specifically, we gathered private (while being alone) and public (while other people are present) positive experiences with technology and compared need fulfillment and affective experience. In addition, we asked participants to imagine a change in context (from private to public or public to private) and to report the impact of this change on experience. Results support the idea of particular social needs, such as relatedness and popularity, which are especially relevant and better fulfilled in public than in private contexts. Moreover, our findings show that participants experience less positive affect when imaginatively removing the present others from a formerly public interaction, i.e., when they imagine performing the same interaction but without the other people present. Overall, this underlines the importance of social context for Human-Computer Interaction practice and research. Practical implications relate to product development, e.g., designing interactive technologies that can adapt to context (changes) or allow for context-sensitive interaction sets. We discuss limitations related to the experimental exploration of social context, such as the method of data collection, as well as potential alternatives to address those limitations, such as diary studies.

## Introduction

Technologization of our everyday lives progresses rapidly. Smartphones, laptops, wearables, and the like accompany us everywhere. Together with their human users, they co-constitute the socio-technical ecosystems we live in (i.e., “mutual constitution” of humans and technologies, Sawyer and Jarrahi, 2014). We are constantly surrounded by technology and permanently interact with it. One major individual outcome of this interaction is experiences, that is, meaningful and enjoyable moments (i.e., wellbeing) mediated through technology use (User Experience, UX; Experience Design, see Hassenzahl, 2010). The present study explores how subjective wellbeing is made through technology (e.g., Desmet and Hassenzahl, 2012; Calvo and Peters, 2014). The user experience of an interactive product is highly context-dependent. It is an ever-changing result of the interplay between the user, technology (i.e., devices), other individuals, and the environment as a whole (Forlizzi and Ford, 2000; for a conceptual distinction from usability, see Hassenzahl and Tractinsky, 2006; Law et al., 2009; Cruz et al., 2015). Thus, it is necessary to consider social dynamics and contextual factors when developing interactive products – especially if they are meant for use in public, such as many mobile devices. For example, imagine you are waiting at a crowded bus station and want to check if there is news regarding the arrival time of your already delayed bus. How would it feel like to use the voice assistant of your smartphone in that situation? Being watched by two people standing close to you, would you prefer using the keyboard of your smartphone instead? Would there be a change in your feelings, thoughts, or behavior if it was only you waiting for the bus? A previous study (Easwara Moorthy and Vu, 2015) on the effect of public vs. private usage contexts showed a higher willingness to use voice assistants in private than in public but showed no difference in preferences regarding keyboard use. This example is only one of many, which demonstrate that social context plays an important role in shaping the way people interact with technology and how they experience technology (e.g., Roto, 2006; Rico and Brewster, 2010; Ens et al., 2015; Grubert et al., 2016; Sergeeva et al., 2017). Interestingly, technology is often insensitive to differences in social context, which is also why Human-Computer Interaction (HCI) experts identify “Human-Environment Interactions” as one of “Seven HCI Grand Challenges” (Stephanidis et al., 2019).

Since social context, i.e., present others, is likely to impact technology experience and use, a better understanding of this influence is necessary. Therefore, the present study addresses the question of how positive experiences with technology differ between private and public contexts. In the following, we give a brief overview of the theoretical concepts of social context and technology-mediated experiences, with an emphasis on the relevance of psychological need satisfaction and subjective wellbeing. We then present an empirical examination of the effect of the presence of other people on need fulfillment and affect. In doing so, we offer deep insights into the emergence of technology-mediated experiences and confirm the existence of social needs, i.e., needs that are more relevant in public contexts. Since we assess a broad spectrum of psychological needs, we draw a more complete picture of technology interactions than previous studies (e.g., Hassenzahl et al., 2010; Tuch et al., 2013; Peters et al., 2018). In conclusion, we discuss the reported observations and implications for further research, product development, and design. Overall, our study results support a positive, need-based approach on designing meaningful (public) interaction experiences.

## Theoretical Background

### Social Context in HCI

In HCI research, Goffman (1959) framework is a popular and widely used theoretical framework when it comes to describing and analyzing how people experience technology in social contexts (e.g., Campbell, 2007a; Dalsgaard and Hansen, 2008; Rico and Brewster, 2010; Colley et al., 2020). Goffman depicted a dramaturgical interpretation of social life, comparing social interaction to theater. According to him, all public action can be understood as performance. People try to manage the impression they make on others by acting in a certain way. Analogous to actors on stage, people play roles to fit the expected social context. More specifically, how people present themselves depends on the audience, the context, and the expectations of their audience’s reactions. Consequently, technology interactions in public spaces should account for the presence of others, i.e., actual or potential spectators, to allow for a pleasant “performance.” Present others can take up different roles depending on their interaction with the system or relationship with the user (e.g., Wouters et al., 2016; Gentile et al., 2017). However, in the present study, social context, i.e., the others present, is not further defined and only general distinction is made between public (someone is present) and private (no one is present) contexts.

The importance of social context and its alleged impact on people’s experience and behavior is widely acknowledged. However, there has been little systematic research on experience-oriented aspects of interactive products explicitly taking contextual factors and social dynamics into account (e.g., Ross and Wensveen, 2010; Lenz et al., 2014). Thus, an adequate theoretical model that includes social context when describing or predicting positive technology use and experience is still missing.

### Positive Technology-Mediated Experiences (in Public)

Previous research on positive technology-mediated experiences understands it as positive affectivity, which emerges from the fulfillment of psychological needs (Sheldon et al., 2001; Hassenzahl et al., 2010). In line with the central role of psychological needs for wellbeing in general (Ryan and Deci, 2000), the notion of psychological needs as a source of positive experiences has a tradition in the field of HCI and UX (e.g., Hassenzahl, 2008a; Hassenzahl et al., 2010; Hassenzahl and Diefenbach, 2012; Tuch et al., 2013; Peters et al., 2018). In fact, any positive experience with technology can usually ultimately be traced back to the fulfillment of a psychological need (Hassenzahl and Diefenbach, 2012). Thus, need fulfillment is understood as a main source of positive experiences with interactive products. Positive design approaches acknowledge the key role of psychological needs and take the users’ experiences to the fore.

However, the few approaches that consider technology interactions as socially embedded often treat social context as a potential source of problems – with the goal to avoid disturbing others (Koelle et al., 2020). Accordingly, interactions are designed to be socially acceptable. This social acceptability is “typically defined through negation, or an absence of negative judgment” (Koelle et al., 2020, p. 6). It encompasses both, the way other people perceive the use of a technical device and the way the user does so him- or herself (Montero et al., 2010). A lack of social acceptability could impact the user’s self-perception as well as other people’s perception (Goffman, 1959), influence the overall user experience (Williamson, 2012), and carry the risk of misperceptions (Shinohara and Wobbrock, 2011) and negative judgment through others (Kleinman, 2007; Koelle et al., 2015; Schwind et al., 2018). So, there is no doubt that social acceptability plays an important role when it comes to the development and design of technology for public application. However, research on social acceptability needs to account for the complexity of social context to overcome theoretical shortcomings (Uhde and Hassenzahl, 2021). Thus, social acceptability might be necessary but is not sufficient to create positive experiences due to the negative, problem-driven perspective. Actually, such problem-driven approaches only aim for eliminating problems or reducing unhappiness rather than promoting happiness (Desmet and Pohlmeyer, 2013). In order to design technology that feels good – instead of not bad – to interact with, it is not enough to ensure social acceptability. One must also understand what makes a technology interaction a positive experience in different social contexts. Previous studies on technology experience in public (and private) are a first step by revealing differences in acceptance and perception of interactive products in public vs. private space (e.g., Rico et al., 2010; Pearson et al., 2015; Hsieh et al., 2016; Lopatovska and Oropeza, 2018; Pandey et al., 2021). The current study contributes to this research and explores the source, i.e., the relevant needs, of positive experiences with technology in public and private contexts.

## Research Focus and Hypotheses

All in all, social context has mostly been neglected in HCI research so far. On the one hand, most design approaches in the fields of HCI, Ergonomics, or Interaction Design predominantly focus on the immediate interaction of user and technology, thereby downplaying the impact of social context. On the other hand, social context (if it is even considered) is yet conceptualized mainly from a restricted, problem-oriented perspective. Our study aims to expand this view on social context. Besides a focus on preventing problems in public interactions, we also consider present others as a potential source for the creation of positive experiences. Hereby, the main emphasis is less on technology type but rather on general, context-specific requirements. The present study explores positive experiences with technologies in public and private contexts and identifies potential differences regarding fulfilled psychological needs. By this means, we reveal whether and how interactions with all kinds of interactive products are shaped by the social context, i.e., absence (private context) or presence (public context) of others. In the following sections, we derive specific hypotheses regarding need fulfillment and affect in private vs. public contexts and highlight our study’s advancements beyond previous research.

Previous studies (Hassenzahl et al., 2010, 2015) already scrutinized the relationship between technology use and need fulfillment on positive affect for social and non-social experiences. In both studies, the presence or absence of other people was associated with differences in need fulfillment. Specifically, Hassenzahl et al. (2010) found that relatedness fulfillment was higher in public situations, i.e., other people had been explicitly mentioned. In contrast, competence, security, and meaning fulfillment were lower in public compared with private situations. Hassenzahl et al. (2015) found relatedness and popularity to be fulfilled to a greater extent when at least one other person was present (i.e., social situations) while meaning was fulfilled to a lower degree. In both studies, however, the presence of other people was not experimentally controlled for, so causal inferences cannot be drawn. Thus, we conduct an experimental between-subjects manipulation of social context to directly compare need fulfillment in public vs. private. In line with Hassenzahl et al. (2010), we hypothesize that need fulfillment for relatedness and popularity is higher in public than in private contexts (H1a and H1b).

H1: Need fulfillment is higher in public compared to private contexts for,

H1a: Relatedness (between-subjects comparison).

H1b: Popularity (between-subjects comparison).

Goffman (1959) described interacting in public as a performance and that people strive to create a specific impression in the minds of others. Therefore, we suggest that in situations with fewer “external forces” (here: other people), the need fulfillment of autonomy is higher than in public contexts. Moreover, Hassenzahl et al. (2010) showed that need fulfillment for competence, security, and meaning is lower in social experiences than in non-social experiences. Accordingly, we hypothesize a lower need fulfillment for competence, security, and autonomy in public compared to private contexts (H2a, H2b, and H2c).

H2: Need fulfillment is lower in public compared to private contexts for,

H2a: Competence (between-subjects comparison).

H2b: Security (between-subjects comparison).

H2c: Autonomy (between-subjects comparison).

In the previous studies (Hassenzahl et al., 2010, 2015), comparisons between social vs. non-social context included various types of experiences; participants reported interactions with different products and in different contexts, and thus, confounding effects cannot be excluded. In order to control for that in our study, we implemented a study design which allowed for within-subject comparisons of the very same interaction in a public and private context. Thus, we can compare participants’ perceptions and evaluations of the same technology interaction in different social contexts, i.e., when other people are present and no one is around. We hypothesize a causal relationship between the presence of others and need fulfillment, specifically, a positive effect for relatedness and popularity (H3a and H3b) and a negative effect for autonomy (H4a).

H3: Need fulfillment is higher for positive experiences in public context than for the same technology interaction without the other people for,

H3a: Relatedness (within-subject comparison).

H3b: Popularity (within-subject comparison).

H4: Need fulfillment is higher for positive experiences in private context than for the same technology interaction with other people present for,

H4a: Autonomy (within-subject comparison).

Furthermore, we expect that context changes, i.e., a modification of social context, have a negative effect on positive affect as well. Since a change of social context presumably leads to lower need fulfillment and need fulfillment is associated with positive affect (Hassenzahl et al., 2010), positive affect should decrease when social context is modified. More specifically, if positive experiences in public mainly arise from need fulfillment of social needs (i.e., relatedness and popularity) and in private contexts from a feeling of autonomy, a switch of contexts should, in turn, lead to lower positive affect (H5a and H5b). However, support for hypotheses on need fulfillment (H3a, H3b, and H4a) has to be found first as a prerequisite for the corresponding affect alterations.

H5: Positive affect is lower in a modified social context.

H5a: Positive affect is lower when removing present others from originally public interactions.

H5b: Positive affect is lower when adding other people to originally private interactions.

## Materials and Methods

The study was pre-registered on the Open Science Framework prior to data collection and analysis. It was realized *via* an online questionnaire and announced as a study on the subjective experience of technology interactions in public and private contexts. All materials were presented in German.

### Participants

Overall, 198 participants who were recruited *via* university mailing lists, snowball sampling, and social media platforms completed the survey. After a first screening of their answers, 14 individuals were excluded from the study on the basis of missing data (e.g., answered central questions with “X”) or an experience that obviously did not fit our criteria (e.g., indicated that “nobody” was present although they had been instructed to recall an interaction occurring in public context). The 184 participants (67.4% female, 32.1% male, and 0.5% diverse) were aged 18 to 71years (*M*=27, *SD*=26.30). As an incentive for their participation, four gift coupons of 25 euros were raffled among all participants. Besides, students could register their participation for course credit. The preconditions for participation were a good knowledge of German.

### Procedure

In the present study, we asked participants to evaluate technology experiences and systematically varied the configuration of the situations through short text vignettes. More specifically, we varied the factors “social context” (public vs. private context, varied between-subjects) and “experience type” (recalled vs. imagined, varied within-subjects), see Table 1.

**Table 1:** Experimental conditions and corresponding instructions to elicit and modify experiences.

Condition^a^	Group^b^	Vignette	Text
pub→prv	recalled public	vignette 1a	Please take a moment to recall a specific, positive experience in which technology in the broadest sense was involved. This should be an experience in which you interacted with a technical product, which one is up to you (e.g., cell phone, robot, food processor, e-scooter, etc. - really any kind of electronics or technology). The interaction should have taken place in public, i.e., one or more other persons were present. Thus, above all it’s important that you had company during your technology interaction and that you experienced this interaction as positive. The other person(s) may also have interacted with the technology, or may have just been present
prv→pub	recalled private	vignette 1b	Please take a moment to recall a specific, positive experience in which technology in the broadest sense was involved. This should be an experience in which you interacted with a technical product, which one is up to you (e.g., cell phone, robot, food processor, e-scooter, etc. - really any kind of electronics or technology). The interaction should have taken place in private, i.e., no other person was present. Thus, above all it’s important that you did not have company during your technology interaction and that you experienced this interaction as positive
pub→prv	imagined private	vignette 2a	Now please try to imagine that no one else would have been present during the product interaction you described earlier, so you would not have had any company. In some situations, this may seem strange or difficult to imagine because another person was directly involved in the original product interaction. Nevertheless, please try to place yourself in the modified situation as best you can
prv→pub	imagined public	vignette 2b	Now please try to imagine that additionally someone else had been present during the product interaction you described earlier, so you would have had company. In some situations, this may seem strange or difficult to imagine. Nevertheless, please try to place yourself in the modified situation as best you can

a *n=96 (pub→prv condition), n=88 (prv→pub condition)*.

b *Each group comprises a specific combination of factor levels of “social context” and “experience type.”*

**Figure 1:**
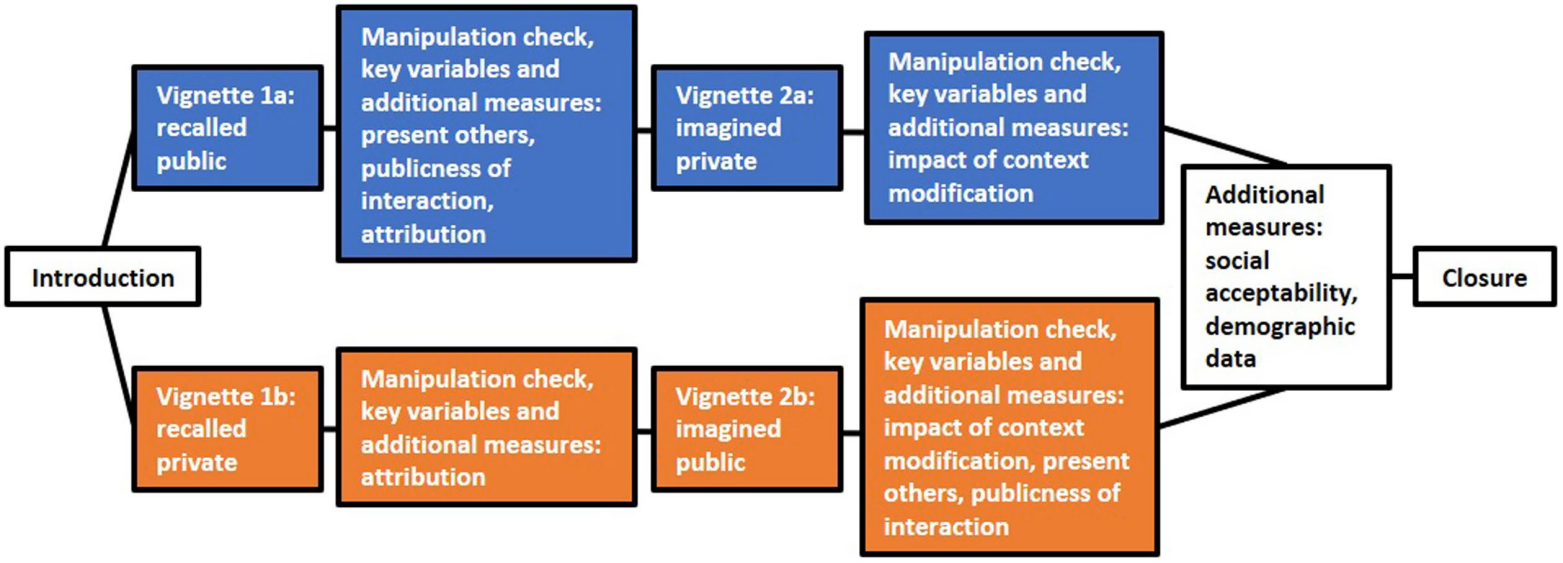
Procedure of the study. The same selection of items was presented to participants of both experimental conditions (in a different sequence). Key variables = need fulfillment and affect.

Participants were randomly assigned to one of the two experimental conditions, i.e., pub→prv condition (“recalled public interaction, followed by imagined private interaction”) or prv→pub condition (“recalled private interaction, followed by imagined public interaction”) after reading an introduction and giving consent agreement. Depending on which group they were in, we instructed participants to recall and describe either a positive technology interaction in *public* or *private* (see Table 1 for the detailed instructions). Participants were asked to provide positive experiences with an interactive product in the broadest sense, such as smartphones, kitchen devices, or e-scooters. After having described the experience, participants evaluated their recalled (public or private) experiences. Subsequently, participants were instructed to reimagine their reported experience in the opposite context. Thus, participants who recalled an experience in public context were now asked to imagine the same experience in private. Again, they were asked to evaluate the imagined experience. Participants rated the recalled and imagined experiences with regard to a variety of measures: affect, need fulfillment, attribution (i.e., what causes the positive experience), relationship to present others, other people’s role, publicness of interaction, impact of context modification (from public to private or private to public), and overall social acceptability of interaction (see “Measures”). Finally, participants in both experimental conditions provided some demographic information (gender, age, and occupation). Except for affect and needs which were assessed after each vignette, i.e., two times, all measures were acquired once. In both experimental conditions (pub→prv condition and prv→pub condition), the same selection of items was presented to participants – in a different sequence. The study procedure is visualized in Figure 1.

### Experience Reports

Participants were instructed with the following text to describe their positive technology interactions: “Please report your product experience as accurately and in detail as possible, trying to be as specific as possible. You can use as many words as you like. Outsiders should be able to easily understand your experience with the help of this description.” Participants’ experience reports were collected with the help of an open text question. Overall, 96 participants (52%) were assigned to the public condition and, consequently, recalled and described a positive experience in public. Another 88 participants reported a positive experience in private. Participants were further asked to specify the location of the experience by choosing one of eight options (i.e., in my own home, in the home of friends or acquaintances, at work, in a public building or in a stranger’s home, on a (motor)bike/in a car/bus/train/plane, in the street or another public space, in a natural setting, and other; selection is based on Scherer et al., 2001). The coding process for the open text question consisted of two steps. First, categories were defined for the type of product (e.g., smartphone) and the interaction’s main function (e.g., entertainment). In a second step, two independent coders were asked to categorize the participants’ reports according to the defined categories (product types: Krippendorff’s *α*=0.73; function: Krippendorff’s *α*=0.73). Multiple assignments were allowed in both cases.

### Measures

Need fulfillment and affect are the two key variables used for testing the hypotheses and were assessed two times in both conditions, once after each experience report. This resulted in four measurements for affect and need fulfillment, respectively. Therefore, internal consistencies of the key variables are given in ranges in the following. Additional measures on attribution, social acceptability, present other(s), shape of interaction, and contextual setting were measured once per condition, i.e., there are two measurements for the whole sample. Internal consistencies for the additional measures are provided separately for both experimental conditions.

#### Affect

Most definitions of wellbeing assume an affective component (e.g., Diener et al., 1985; Hassenzahl et al., 2010; Martela and Sheldon, 2019). Thus, we administered the Positive and Negative Affect Schedule (PANAS; Watson et al., 1988; German translation by Krohne et al., 1996) to assess this facet of subjective wellbeing. It has been found to be a reliable and valid measure (Crawford and Henry, 2004) and is well established in HCI and UX research (e.g., Hassenzahl, 2008b; Partala and Kallinen, 2012; Anderson et al., 2017; Klapperich et al., 2020). Another reason for choosing PANAS over other measures was the fact that it allows for capturing positive and negative emotions separately. Consequently, PANAS was selected as the emotion assessment method for the current research. The scale consists of 20 verbal descriptors of different facets of affective experiences (e.g., scared, nervous, inspired, and proud) and covers positive as well as negative valence of affect through two subscales with 10 items each. Participants were asked to rate how well each of these 20 attributes described their affect during the respective experience on a 5-point scale ranging from 1 (not at all) to 5 (extremely). Even though we explicitly asked participants to provide positive experiences only, we included negative affect for exploratory analyses. Positive affect (PA) and negative affect (NA) scores were calculated in the present study by averaging the responses to the 10 affect descriptors for each valence. Internal consistency of positive and negative affect was good (PA: *α*=0.82–0.83; NA: *α*=0.75–0.92; see Table 2). Inter-scale correlations were mainly small and insignificant (*r*=−0.01–−0.38).

**Table 2:** Inter-correlation of key variables need fulfillment and affect for recalled (in brackets: imagined) experiences. The diagonal (bold) displays internal consistencies (Cronbach’s alpha) for each scale.

Measures	1		2		3		4		5		6		7	
	Pub	Prv	Pub	Prv	Pub	Prv	Pub	Prv	Pub	Prv	Pub	Prv	Pub	Prv
1. Relatedness	**0.91 (0.93)**	**0.92 (0.95)**												
2. Popularity	0.26^*^ (0.66^**^)	0.58^**^ (0.60^**^)	**0.67 (0.83)**	**0.77 (0.81)**										
3. Competence	0.00 (0.19)	0.24^*^ (0.34^**^)	0.42^**^ (0.34^**^)	0.42^**^ (0.62^**^)	**0.74 (0.87)**	**0.73 (0.79)**								
4. Security	0.15 (0.32^**^)	0.34^**^ (0.33^**^)	0.60^**^ (0.52^**^)	0.37^**^ (0.41^**^)	0.40^**^ (0.41^**^)	0.49^**^ (0.50^**^)	**0.65 (0.71)**	**0.68 (0.79)**						
5. Autonomy	0.24^*^ (0.22^*^)	0.41^**^ (0.51^**^)	0.57^**^ (0.38^**^)	0.56^**^ (0.63^**^)	00.33^**^ (0.51^**^)	0.34^**^ (0.52^**^)	0.38^**^ (0.50^**^)	0.46^**^ (0.64^**^)	**0.70 (0.81)**	**0.71 (0.72)**				
6. Positive Affect	0.16 (0.23^*^)	0.17 (0.26^*^)	0.32^**^ (0.43^**^)	0.43^**^ (0.52^**^)	0.45^**^ (0.52^**^)	0.51^**^ (0.66^*^ ^*^)	0.33^**^ (0.33^**^)	0.38^**^ (0.43^**^)	0.34^**^ (0.53^**^)	0.39^**^ (0.43^**^)	**0.83 (0.89)**	**0.82 (0.88)**		
7. Negative Affect	−0.10 (0.11)	0.00 (−0.03)	0.01 (0.14)	0.03 (0.02)	0.15 (0.09)	−0.09 (0.04)	−0.07 (0.04)	−0.20 (−0.21^*^)	−0.17 (−0.08)	−0.15 (−0.21^*^)	−0.02 (−0.03)	−0.06 (−0.22)	**0.75 (0.85)**	**0.75 (0.92)**

*Pub, pub→prv condition (n=96); Prv, prv→pub condition (n=88)*;

* *p< 0.05*;

** *p< 0.01*.

#### Need Fulfillment

Fulfillment of different needs was measured with the questionnaire of Sheldon et al. (2001; German translation by Diefenbach and Hassenzahl, 2010) except for self-esteem which we consider an outcome of need fulfillment rather than a need itself (Hassenzahl et al., 2010). General need fulfillment was computed by averaging the scores of all nine needs. Participants were asked to assess the following needs on a 5-point scale ranging from 1 (not at all) to 5 (extremely): relatedness (e.g., Item 2: “I felt close to people who are important to me”), popularity (e.g., Item 1: “I felt like a person whose opinion is valued by others”), competence (e.g., Item 1: “I felt that I was successfully completing difficult tasks”), security (e.g., Item 2: “I felt that I have a comfortable set of routines and habits”), autonomy (e.g., Item 2: “I felt that things can be done in my own way”), luxury (e.g., Item 3: “I felt that I got plenty of money”), stimulation (e.g., Item 3: “I felt that I was experiencing new sensation and activities”), physical striving (e.g., Item 2: “I felt that my body was getting just what it needed”), and meaning (e.g., Item 1: “I felt I was becoming who I really am”) – with three items each. However, we only used the first five needs for hypotheses testing (i.e., relatedness, popularity, competence, security, and autonomy) and included the latter four in exploratory analyses.

Overall, the internal consistency (Cronbach’s alpha) was acceptable for most of the needs with exception of popularity, luxury, and security. Since the criterion of 0.70 (according to Nunnally, 1975) has not been reached in both conditions, we performed item reduction for each respective scale. Thereby, we achieved a substantial improvement of Cronbach’s alphas for luxury [*α*=0.73–0.81; Item 2 (“I felt that I have nice things and possessions”) excluded] and security [*α*=0.65–0.79; Item 3 (“I felt safe from threats and uncertainties “excluded)], but not for popularity (*α*=0.67–0.83). Cronbach’s alphas and scale inter-correlations of the needs relevant for hypotheses testing are in Table 2.

#### Additional Measures

##### Manipulation Check

After each vignette, we first asked participants to indicate how well they could immerse themselves in the situation using a 5-point scale (1=not at all and 5=extremely). Besides, we offered participants to declare if they had difficulties with the imagined context modification the second vignette asked for. Experience reports of participants with a score lower than three for the manipulation checks were examined and excluded if necessary. Finally, our sample includes 14 participants with such low scores.

##### Attribution

We asked participants to assess the extent to which the product caused the experience on a 5-point scale (1=very small and 5=very large).

##### Social Acceptability

We used two questions, adapted from Koelle et al. (2018), to assess social acceptability of the technology interaction. Participants were asked to indicate “How comfortable would you feel performing this product interaction in a public setting?” and “How acceptable would it be to perform this product interaction in public?” on a 5-point scale (1=not at all and 5=extremely). Internal consistency (Cronbach’s alpha) was acceptable to good (pub→prv condition: *α*=0.83; prv→pub condition: *α*=0.79).

##### Present Others

We used two questions to clarify the relationship with and involvement of person(s) present during public experiences. First, participants indicated their relationship by selecting one or more options from a list of different categories: “nobody,” “a friend or partner,” “a colleague or acquaintance,” “several friends or acquaintances,” “one or more unspecified persons,” “large crowd,” and “other” (selection is based on Scherer et al., 2001). Second, they were asked about the involvement of those present others in the interaction itself on a 5-point scale (1=passive and 5=active).

##### Publicness of Interaction

We created four items to measure participants’ perceived publicness of technology interaction: “I (would have) felt like I was being watched,” “I (would have) cared what other people might think,” and “During my product interaction, I would have been/was at the center of attention of the other person/s.” Participants indicated their agreement with each item on a 5-point scale (1=not at all and 5=fully). The average of all three scores serves as an indicator of how public the situation was subjectively experienced by each participant. Originally, we created four items; however, inter-item correlations were at least questionable in both conditions. Thus, we excluded one item [“I (would have) experienced the interaction as public”] which leads to a slightly improvement of Cronbach’s alphas (pub→prv condition: *α*=0.59; prv→pub condition: *α*=0.70).

##### Impact of Context Modification

One quantitative and one qualitative question captured the impact of the within-subject manipulation of social context, i.e., when people were added or removed to the recalled experience. First, participants quantified how the interaction would feel like in the modified context compared to the original one by rating the valence on a 5-point scale from “worse” to “better” (with the midpoint indicating no change). Second, participants were supposed to elaborate on the effects of the presence (prv→pub condition) or absence (pub→prv condition) of others on their feelings, thoughts, and behavior in an open-ended answer.

## Results

All analyses were conducted with SPSS (IBM Statistics Version 26).

### Experience Reports

**Figure 2:**
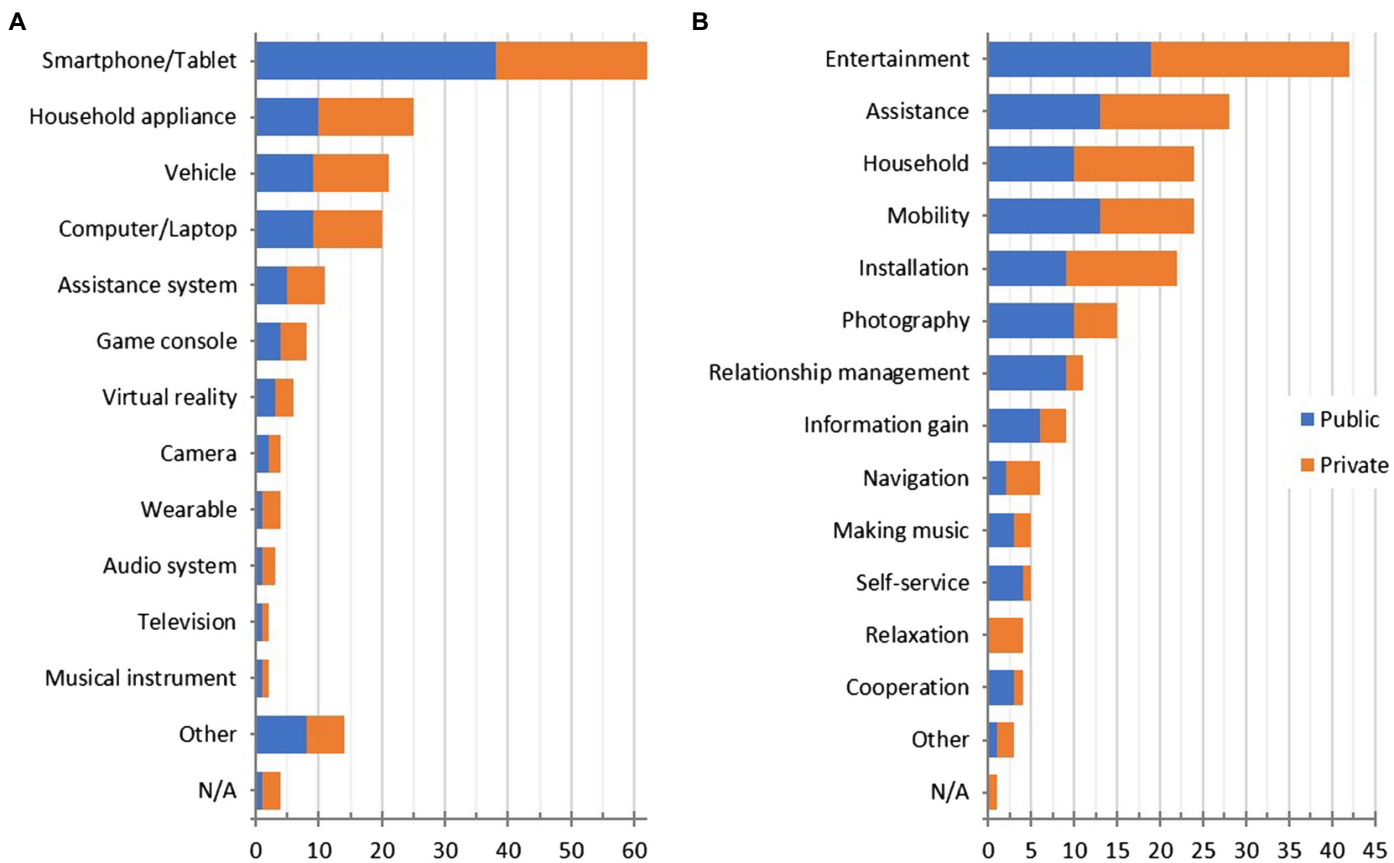
Categorization of recalled experiences in public and private context by **(A)** product types and **(B)** function of interactions. Absolute frequency of all categories in total sample.

An example of a technology experience (i.e., a recalled interaction in public context) described in the pub→prv condition reads as follows, “*I was at Legoland with my family. There were robot arms that whirled you through the air, similar to a roller coaster ride. However, you could choose the movements of the arm yourself*.” The other half of the sample recalled positive technology interactions in private context, i.e., while being alone. An exemplary report from the prv→pub condition was, “*When the new album of one of my favorite artists came out, I immediately listened to it with my Bluetooth headphones and the experience - hearing it for the first time - was indescribable. At that moment I was indeed happy about the technology and digitalization that conquers the whole world nowadays*.” Figure 2 provides an overview of the obtained technology reports.

Participants’ answers regarding locations of recalled technology interactions were quite equally distributed across the provided options. In the pub→prv condition, 19% of the (recalled public) interactions took place “on the streets or another public place,” 18% “in my own home,” 14% “in the home of friends or acquaintances,” and 14% “in a natural setting.” However, a different picture emerged in the prv→pub condition (for interactions in private); most of the participants’ recalled experiences (67%) took place in their own homes. Participants’ answers to the question of how their thoughts, feelings, or behavior change through the modification of context provided further insights into the effect of the presence or absence of others. For example, when a participant in the pub→prv condition was asked to imagine their originally public interaction without other people being present, they described the change as follows, “*I can try everything as long as I want, have no stress, can take the time I need and I am relaxed. But I also have a smaller sense of achievement when no one is there to watch*.” A representative example for the prv→pub condition is, *“Alone, you could do everything according to your own wishes and ideas and be proud of having actually done it alone, however, there is also no one with whom you can share the joy. Conversely, in the presence of another person, you have a shared sense of achievement and can rejoice together. However, you do not feel quite as free in your application and may adapt to the other person’s opinion or feel more pressure because you want it* [the product] *to work when someone is watching you.”*

In order to check if public and private contexts were inherently different with regard to positive affect, we conducted a one-way ANOVA. Results showed no effect of “social context” (public vs. private context) on positive affect [*F*(1, 182)=0.41, *p*=0.523]. Thus, there were no systematic differences to be further considered.

### Hypotheses Testing

One-way ANOVAs were conducted to allow between-subjects comparisons of recalled experiences, i.e., actually experienced interactions, with regard to social context, i.e., public vs. private context. We assumed an effect of “social context” on need fulfillment for relatedness and popularity; particularly, we expected higher scores for relatedness (H1a) and popularity (H1b) when participants recalled experiences in public compared to private contexts. The opposite was assumed for competence, security, and autonomy (H2a, H2b, and H2c). All reported effect sizes were calculated using the partial eta square ($\eta_{p}^{2}$), with 0.01, 0.06, and 0.14 considered small, medium, and large effects, respectively (Lakens, 2013). Table 3 shows means and standard deviations of the relevant five needs and positive affect (PA), and results of between- and within-subject comparisons.

**Table 3:** Means (*M*) and standard deviations (*SD*) of need fulfillment and significance of statements on between-subjects and within-subject group differences.

Measure	Experimental Group								Analysis	*F* ^c^	$\eta_{p}^{2}$
	recalled public^a^		recalled private^b^		imagined public^b^		imagined private^a^				
	*M*	*SD*	*M*	*SD*	*M*	*SD*	*M*	*SD*			
Relatedness	3.28	1.30	2.05	1.24	2.98	1.37	1.63	1.03	H1a	43.24^***^	0.19
									H3a	136.90^***^	0.59
Popularity	2.83	1.03	2.18	1.00	2.64	1.09	1.93	0.99	H1b	19.11^***^	0.10
									H3b	81.67^***^	0.46
Competence	3.01	1.02	3.15	0.98	3.03	1.01	2.84	1.18	H2a	0.86	0.01
Security	2.64	1.12	2.92	1.14	2.80	1.20	2.38	1.22	H2b	2.82	0.02
Autonomy	2.97	0.97	3.16	0.96	2.74	1.02	2.77	1.16	H2c	1.78	0.01
									H4a	20.75^***^	0.19
Positive Affect	3.69	0.66	3.63	0.68	3.47	0.80	3.34	0.83	H5	29.21^***^	0.14

a pub→prv condition (*n*=96)

b prv→pub condition (*n*=88)

c *F(1, 182) for hypotheses 1, 2, and 5; F(1, 95) for hypotheses 3; and F(1, 87) for hypothesis 4*

*** *p< 0.001*.

Results supported our first two hypotheses by showing that participants experienced more need fulfillment of relatedness (H1a) and popularity (H1b) in public than private contexts. First, “social context” (public vs. private) had a significant effect on relatedness [*F*(1, 182)=43.24, *p*<0.001, $\eta_{p}^{2}$=0.19] and popularity fulfillment [*F*(1, 182)=19.11, *p*<0.001, $\eta_{p}^{2}$=0.10]. Relatedness was significantly higher for recalled experiences in public (*M*=3.28, *SD*=1.30) compared to private (*M*=2.05, *SD*=1.24). Second, popularity was significantly higher in public context (*M*=2.83, *SD*=1.03) compared to private context (*M*=2.18, *SD*=1.00). In contrast, our hypotheses regarding competence (H2a), security (H2b), and autonomy (H2c) have to be rejected as “social context” had no effect on the need fulfillment of competence [*F*(1, 182)=0.86, *p*=0.354], security [*F*(1, 182)=2.82, *p*=0.095], and autonomy [*F*(1, 182)=1.78, *p*=0.183]. Overall, we found support for relatedness and popularity being social needs, i.e., needs especially relevant for public contexts, but no indication that competence, security, and autonomy could be labeled typical private, non-social needs.

Previous analyses of recalled experiences in public vs. private compared experiences with different products between people. To reduce potential confounding effects, we formulated hypotheses on within-subject comparisons and conducted one-way repeated-measures ANOVAs. Similar to the effect of “social context” in the between-subjects comparison, we expected to find an effect of context modification, i.e., “experience types” (recall vs. imagination), on need fulfillment. More specifically, we hypothesized that fulfillment of relatedness, popularity, and autonomy differs for “experience types” with higher values for recalls, i.e., interactions that participants actually experienced, than for imagination, i.e., when adding or removing others to/from the recalled experiences. Regarding relatedness (H3a) and popularity (H3b), the conducted ANOVAs revealed a significant main effect for “experience type” [relatedness: *F*(1, 95)=136.90, *p*<0.001, $\eta_{p}^{2}$=0.59; popularity: *F*(1, 95)=81.67, *p*<0.001, $\eta_{p}^{2}$=0.46]. Thus, in the pub→prv condition, the context modification, i.e., imaginatively removing present others, led to a decrease in need fulfillment of relatedness and popularity as need scores were lower in imagined (relatedness: *M*=1.63, *SD*=1.03; popularity: *M*=1.93, *SD*=0.99) compared to recalled (relatedness: *M*=3.28, *SD*=1.30; popularity: *M*=2.83, *SD*=1.03) experiences.

Regarding autonomy (H4a), one-way repeated-measures ANOVA revealed a significant effect of “experience type” [*F*(1, 87)=20.76, *p*<0.001, $\eta_{p}^{2}$=0.19] in the prv→pub condition as fulfillment of autonomy differed for recalled (*M*=3.16, *SD*=0.96) and imagined (*M*=2.74, *SD*=1.02) experiences. More specifically, autonomy was fulfilled less when imaginatively adding other people to a formerly private interaction.

Overall, within-subject comparisons (between recalled and imagined experiences) revealed a positive causal relationship between the presence of other people and need fulfillment for relatedness and popularity, and a negative one for autonomy. Since need fulfillment and positive affect are linked, we expect positive affect to be lower in imagined compared to recalled experiences. Thus, on the one hand, we suggested that experiences in public context are perceived less positively when no others are present (H5a). On the other hand, we proposed a decrease in positive affect when other people are added to formerly private interactions (H5b). Prerequisites (H3a, H3b, and H4a) are fulfilled as need fulfillment decreases when modifying social context through the imaginative addition (or removal) of other people. Consequently, we conducted a two-way mixed ANOVA and results showed significant main effect of “experience type” for positive affect [*F*(1, 182)=29.21, *p*<0.001, $\eta_{p}^{2}$=0.14]. In addition, we found a significant interaction between “experience type” and “social context” [*F*(1, 182)=4.24, *p*=0.041, $\eta_{p}^{2}$=0.02] and no significant main effect of “social context” [*F*(1, 182)=0.10, *p*=0.752]. More specifically, overall positive affect scores were higher in recalled experiences (pub→prv condition: *M*=3.69, *SD*=0.66; prv→pub condition: *M*=3.63, *SD*=0.68) compared to the modified versions, where people were imaginatively removed or added (pub→prv condition: *M*=3.34, *SD*=0.83; prv→pub condition: *M*=3.47, *SD*=0.80). Thus, hypotheses 5a and 5b were supported.

### Exploratory Analyses

We conducted further exploratory analyses to gain deeper insights on the underpinnings of positive technology experiences in public space and the impact of present others.

#### Need Profiles

**Figure 3:**
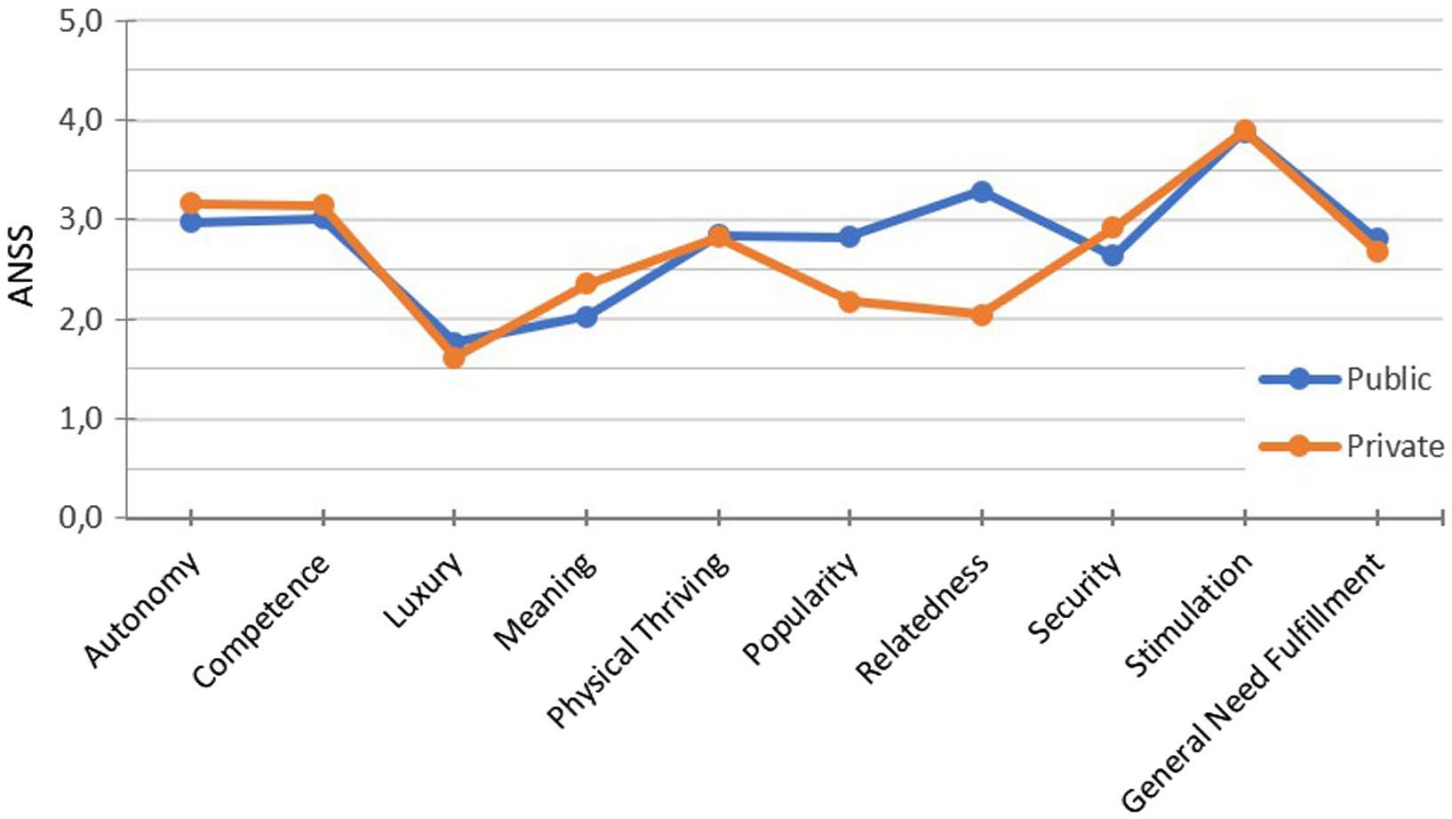
Needs profiles for recalled experiences in public and private context. ANS, average need fulfillment score.

Initially, we extended our focus to all nine measured needs to draw a more complete picture of the need fulfillment in technology experience. We created need profiles by comparing the need fulfillment for the initially given (i.e., recalled) public and private experience (see Figure 3). The detected need profiles are quite similar and show no significant differences besides those in relatedness and popularity reported above. An additional analysis of general need fulfillment showed that in both conditions general need fulfillment was equally high (public: *M*=2.81, *SD*=0.65; private: *M*=2.68, *SD*=0.71).

#### Needs and Affect

Furthermore, we explored relationships between our key variables, i.e., need fulfillment and affect. To this end, we pooled the recalled and imagined experiences, resulting in a dataset of 368 experiences (two for each of the 184 participants). Means, standard deviations, and Pearson correlations of the respective variables are illustrated in Table 4. Our analyses showed that high values for all needs are associated with high values for PA, *r*(366)=0.23–0.69. In contrast, NA only correlated negatively with two needs: autonomy [*r*(366)=−0.17, *p*<0.01] and physical thriving [*r*(366)=−0.17, *p*<0.01]. However, such correlations can be considered rather low, especially when compared to the strong correlations between PA and general need fulfillment, *r*(366)=0.61, *p*<0.001.

**Table 4:** Means (*M*), standard deviations (*SD*), and correlations of need fulfillment, positive, and negative affect.

Measures	*M*	*SD*	1	2	3	4	5	6	7	8	9	10	11	12
1. Autonomy	2.91	1.04	–											
2. Competence	3.00	1.06	0.44^**^	–										
3. Luxury	1.66	0.89	0.28^**^	0.13^*^	–									
4. Meaning	2.10	1.07	0.68^**^	0.43^**^	0.33^**^	–								
5. Physical Thriving	2.69	1.16	0.56^**^	0.40^**^	0.22^**^	0.61^**^	–							
6. Popularity	2.39	1.09	0.49^**^	0.43^**^	0.30^**^	0.48^**^	0.45^**^	–						
7. Relatedness	2.49	1.41	0.29^**^	0.18^**^	0.13^*^	0.33^**^	0.36^**^	0.58^**^	–					
8. Security	2.68	1.18	0.50^**^	0.45^**^	0.33^**^	0.50^**^	0.52^**^	0.46^**^	0.27^**^	–				
9. Stimulation	3.66	1.06	0.45^**^	0.42^**^	0.25^**^	0.40^**^	0.44^**^	0.37^**^	0.22^**^	0.23^**^	–			
10. General Need Fulfillment	2.62	0.75	0.77^**^	0.63^**^	0.46^**^	0.78^**^	0.76^**^	0.76^**^	0.59^**^	0.71^**^	0.61^**^	–		
11. Positive Affect	3.53	0.76	0.45^**^	0.54^**^	0.23^**^	0.36^**^	0.47^**^	0.43^**^	0.23^**^	0.37^**^	0.69^**^	0.61^**^	–	
12. Negative Affect	1.30	0.45	−0.17^**^	0.05	0.06	0.02	−0.17^**^	0.04	−0.01	−0.10	−0.06	−0.06	−0.12^*^	–

*N=368*;

* *p< 0.05*;

** *p< 0.01*.

#### Additional Factors

In order to reveal differences in how socially acceptable interactions in public and private contexts are, we conducted an exploratory one-way ANOVA. Social acceptability ratings differed between private (*M*=3.23, *SD*=1.15) and public context (*M*=4.01, *SD*=0.97; *F*(1, 182)=24.70, *p*<0.001, $\eta_{p}^{2}$=0.12). Thus, people seem to perform less socially acceptable technology interactions in private compared to public situations. In addition, we conducted an exploratory analysis of bivariate correlations between social acceptability and the key variables to investigate if and how social acceptability is linked to need fulfillment and affect. Results showed a strong correlation with NA but not PA, such that higher values for social acceptability come with lower values for NA, *r*(182)=−0.24, *p*<0.01.

Furthermore, participants were asked to indicate the valence of context modification, i.e., if they perceive the same interaction as “better” or “worse” when adding or removing others. Results of exploratory one-way ANOVAs revealed differences between the two experimental conditions, *F*(1, 182)=13.70, *p*<0.001, $\eta_{p}^{2}$=0.07. Apparently, participants in the prv→pub condition assessed the addition of other people to formerly private interactions rather neutral (*M*=2.93, *SD*=1.27). In comparison, participants in the pub→prv condition indicated that the imagined experiences, i.e., formerly public interactions without others being present, would feel worse (*M*=2.30, *SD*=1.04). In fact, only 23.4% of all participants experienced the context modification as improvement, i.e., chose four or five on a 5-point scale, with the majority of these people (32 of 43 participants) reporting imagined experiences in public contexts. More specifically, in the prv→pub condition, 40.9% of the participants indicated that they experienced the imagined presence of others as negative (scoring lower than three on the 5-point scale), whereas 62.5% in the pub→prv condition claimed that their experience was worse when imagining performing the same interaction in private context.

We also assessed how active (vs. passive) present others were in the public interactions and explored if this influenced reported affect. There was a medium positive correlation between the involvement of others in the technology interaction and PA [*r*(182)=0.34, *p*<0.001]; a more active role of present others was correlated with higher PA values for public interactions regardless if it was a recalled or imagined experience. Besides, present others played a more active role in recalled (*M*=3.43, *SD*=1.46) compared to imagined (*M*=2.65, *SD*=1.43) experiences, *F*(1, 182)=13.37, *p*<0.001, $\eta_{p}^{2}$=0.07. Consequently, when participants recalled public interactions, present others were perceived as active in contrast to imagined public interactions in which participants assign them a more passive role.

Additionally, an analysis of the perceived publicness of the interaction showed that people in the pub→prv conditions (*M*=2.76, *SD*=1.05) and the prv→pub conditions (*M*=3.31, *SD*=1.06) rated the publicness of their interactions surprisingly different, *F*(1, 182)=12.49, *p*=0.001, $\eta_{p}^{2}$=0.06. Since a public context implicates the presence of other people during technology interactions, i.e., potential spectators, we expected to find a left-skewed distribution of scores. However, scores were nearly equally distributed over all answer options. In fact, only 17 of 96 participants in the pub→prv condition rated their experiences as strongly public (i.e., values higher than four on a 5-point scale). For participants in the prv→pub condition, this trend was even larger; 39.8% (35 of 88 participants) stated that their imagined experience would feel strongly public to them (i.e., score of four or higher).

## Discussion

The present study aimed to investigate how positive experiences with technologies emerge in (non-)social contexts. In addition to context-dependent differences in need fulfillment, we were interested in the direct impact of present others on users’ subjective experiences. We predicted that there are needs particularly relevant in public context, as well as needs that are fulfilled more in the private context. In fact, we assumed that the fulfillment of such needs is causally linked to the presence or absence of others. Consequently, in the very same interaction, certain needs play a more or less important role depending on social context. Following up on this, we suggested a positive relationship between need fulfillment and positive experience and predicted a lower positive affect for modified contexts, i.e., when people were removed from interactions in public or were added to interactions in private contexts.

Our results support hypotheses 1a, 1b, 3a, and 3b by showing that the fulfillment of relatedness and popularity is higher in recalled public interactions compared to recalled private interactions (H1a and H1b) and when people are imaginatively added to a formerly private situation (H3a and H3b). We could not find support for hypotheses 2a, 2b, and 2c, as we found similar need fulfillment for competence, security, and autonomy in public and private contexts. However, results support hypothesis 4a and show that imaginatively removing people from formerly public situations while performing the same interaction resulted in greater autonomy fulfillment. Therefore, we conclude that there is a category of “social needs” that are particularly relevant in public contexts and whose fulfillment depends on the presence of other people. But on the other hand, there are apparently no equivalent “non-social needs” that specifically pertain to private contexts. Competence and security did not differ between contexts and we could only find inconclusive results for a higher need fulfillment of autonomy when being alone. In line with Hassenzahl et al. (2015), we found context-dependent differences in need fulfillment only for popularity and relatedness but not for security and competence. Thus, we could not replicate the findings of Hassenzahl et al. (2010) showing that competence and security were significantly less salient in social situations. Apparently, the presence of others did not automatically limit the experience of feeling competent, secure, or autonomous. Possible explanations for the absence of these effects are described in the following.

Regarding competence, one could argue that this need also plays a central role in public contexts as people strive to give the impression of being capable. Remember our example from the introduction, waiting at a bus station while using the voice assistant of your smartphone. In the case of smooth and successful speech interaction, your feeling of competence probably would not be negatively affected by present others. Taking it even one step further, perhaps it would feel good to master the speech interaction precisely because other people are watching you? However, since our data neither showed significant differences between public and private context nor between recalled and imagined experience, one might conclude that the fulfillment of the need for competence is not exclusively arising from social context.

Security does not seem to be mainly relevant for technology interactions in private context either. Participants’ need fulfillment did not differ significantly between the recalled experiences in public and private contexts. Screening of participants’ experience reports suggests that the need for security can also be fulfilled in public contexts as others may support feeling “safe and in control of your life” (Sheldon et al., 2001). For example, one participant described the preparation of coffee with an electronic machine together with their mother as a pleasant ritual. Furthermore, by taking a closer look at the answers of participants in the prv→pub condition on how a modification of social context (i.e., removal of other people) would affect them, some users conceived present people as potential sources of support. Thus, present others do not necessarily make life more unpredictable and could even function as supporters, e.g., when having experience or expertise with the particular technology. Given that only 12.5% of the people attending the technology interactions in public context were unknown persons, the possibility of receiving help from known others might have contributed to feeling secure and thus to similar security fulfillment in recalled private and public situations. This illustrates how complex the influence of “social contexts” can be.

Contrary to our expectation, the need for autonomy was not fulfilled to a higher extent in private contexts. Even though autonomy fulfillment was lower when people were imaginatively added to what was originally a private interaction, it should not be concluded that performing the same interaction while being alone awakes a greater feeling of autonomy compared to being surrounded by others. We can think of the following possible explanations for the contradictory results regarding the feeling of autonomy (H2c and H4a). On the one hand, even when interacting with technology in a public context, the user is still the one in control of the interaction, regardless of whether someone is present. On the other hand, a decrease in autonomy fulfillment could be caused by the experimental manipulation. People might have perceived our instruction to imagine a change to their previously recalled experience as an intrusion of their autonomy, since “external forces or pressure are the cause of (their) action” (Sheldon et al., 2001, p. 339), and therefore assessed the fulfillment of autonomy lower in response. However, social desirability (Grimm, 2010) could serve as a possible explanation as well. Since low need fulfillment for competence, security, and autonomy seems undesirable in social situations, participants could have adapted their response patterns.

Our results support hypotheses 5a and 5b as we detected a decrease of positive affect when context was modified. For example, removing other people from a recalled public interaction led to less positive affect. This second key finding of our study is in line with previous research (e.g., Hassenzahl, 2010), which showed a correlation of social context and need fulfillment as well as a correlation of need fulfillment and positive affect. In our view, the most compelling explanation for the difference in positive affect between recalled public and imagined private interaction is a (missing) compatibility of social context and technology interaction. By showing that the modification of social context, i.e., removal of present others, had a negative impact on the experienced effect of participants, our study stresses the risk of performing an interaction in an “unsuitable” context. For example, imagine using your smartphone to listen to your favorite song after a long day of work. In order to relax, you put on earphones and start dancing and singing along. For some people, the presence of other people might disturb this experience. Results of our exploratory analyses also support this potential explanation; only 23.4% of all participants indicated that the modification of social context would lead to an improvement of their technology experience. Hereby, participants in the prv→pub condition, i.e., imaginatively adding other people to a formerly private interaction, make up the majority of this group. We conclude that the context modification in form of adding people to a private situation was perceived as neutral or even as a potential gain. In contrast, removing present others from public situations was experienced as negative or as a loss.

Another important finding is the (non-)correlation of social acceptability and need fulfillment with experienced affect. General need fulfillment only correlated with positive but not negative affect. But the opposite is true for social acceptability, which correlated with negative but not positive affect. Thus, a lack of social acceptability can be detrimental, but positive experience may arise independently through the fulfillment of needs. We conclude that a conceptual distinction between the preventative social acceptability view and a positive, need-based perspective on designing meaningful technology experiences is reasonable and necessary. Parallels can be drawn with previous research (Hassenzahl et al., 2010) which, inspired by Herzberg (1959) two-factor theory, distinguished “hygiene factors” and “motivators” when it comes to explaining the emergence of positive technology experience. Hassenzahl et al. (2010, p. 359, 361) described motivators as “the product’s perceived ability to create positive experience through need fulfillment” and hygiene factors as instrumental aspects of interactive products “dampening negative affect but not being a source of positive experience in itself.” Moreover, results of exploratory analyses also support the idea of context-interaction fit by revealing higher social acceptability of recalled experiences in public compared to private contexts and linking social acceptability to negative affect. Formerly, public interactions were basically perceived as socially acceptable. But positive private technology interactions turned out to be potentially “unacceptable” when adding others to the situation. Since low social acceptability is associated with negative affect, interacting with an interactive technology insensitive to changes in social context bears the risk of not only receiving negative reactions from others but also experiencing the interaction more negatively oneself.

## Implications For Research and Practice

Taken together, we showed that the sources of positive experience differ systematically depending on the social context (i.e., presence or absence of other people) and that context changes from public to private or vice versa can have a substantial impact on how people experience interactive technologies.

Our results support the causality assumption of the effect of social context on need fulfillment. Thus, they underline the importance of extending theoretical frameworks with a notion of social context that accounts for its positive potential for technology experiences (instead of its constraints). For example, two of the most widely applied models of technology use are Technology Acceptance Model (Venkatesh and Bala, 2008) or Unified Theory of Acceptance and Use of Technology (Venkatesh et al., 2016). However, these models mainly consider subjective norms, social influences, and facilitating conditions of contexts as shaping merely the acceptance of technologies. As we laid out before, these considerations are in line with a negative social acceptability view on context effects, i.e., more of an ought-to than a want-to perspective. This implies an extrinsic motivation to technology use, but the presence of others might also boost users’ intrinsic motivation since those others contribute to the positive user experience. Hornbæk and Hertzum (2017 p. 26) who investigated previous literature on technology acceptance and user experience already stated that the experiential component in HCI is still not well recognized. In accordance with our approach, they emphasized that the concept of psychological needs would help to analyze and understand the motivation for technology adaption and use and criticized that, currently, “accounting for social aspects of use and incorporating them into modeling of experiences (still) seems underdeveloped”. Besides, the finding of social acceptability as a “hygiene factor” (i.e., reducing negative affect but not supporting positive affect) highlights the relevance of a shift in focus to the creation of positive public interactions through deliberate need fulfillment. However, to create a “context-sensitive” model which considers wellbeing as a result of need fulfillment in dynamic social contexts for describing and predicting positive technology experience, further research is needed because social context is more complex than the distinction in private and public context (see “Limitations and Future Research Directions”).

Regarding practical implications, our study emphasizes the importance of considering contextual aspects and psychological needs when designing interactive technologies. For example, design approaches for public space should consider ways to establish relatedness to present others and/or foster a popular impression of the user toward them. Re-designing smartphones by adding a display on their backs allows counterparts or spectators, respectively, to gain insights into what exactly the user is doing when interacting with their smartphone (Jarusriboonchai et al., 2016). By informing or even involving the counterpart in one’s phone activities, a greater feeling of relatedness in user and spectator could be generated (Beukeboom and Pollmann, 2021). Concrete design implications for technologies that address a varying social context could be drawn from, for example, context-aware devices, i.e., systems which are aware of their surroundings and respond intelligently to environment changes (Colley et al., 2016). Interactive products which offer two (or more) “modes” for the context of use depending on its publicness might be good solutions. A simple example is the “silent mode” of smartphones; if users find themselves in a public situation where talking on the phone or even a ringing smartphone would be a distraction or disturbance (for the user or present others), turning on silent mode is a way to avoid these negative effects. However, such solutions rely on users recognizing if an interaction could have negative consequences for themselves or others and adjusting their behavior. This means that there is a dependence on the user’s assessment of the context-interaction fit and willingness (and ability) to react to it. Here, dynamically changing contexts (Dix et al., 2000) and the fact that people sometimes interact with technology, e.g., mobile phones, out of habit or even implicitly (Humphreys, 2005) pose a challenge. In these situations, people may not be aware of when and how their use impacts themselves and others and will not make a deliberate decision for or against an interaction (type). Thus, designers and researchers must face the challenges which arise in the rapidly evolving landscape of interactive technologies and develop adaptive, intelligent products which serve people – users and present others – well (Ens et al., 2015; Grubert et al., 2016). Since our analyses showed that active involvement of present others in participants’ experiences is linked to positive user affect, another practical implication for the design of interactive technologies may lie in the deliberate engagement of spectators in users’ technology interactions – if desired. Previous research on (social) engagement and collaboration *via* interactive products (e.g., Fails et al., 2010; Lucero et al., 2011) can be used as orientation and inspiration for such studies.

## Limitations and Future Research Directions

The present study has several limitations that should be addressed in future research. First, some limitations need to be acknowledged with regard to the research methods. With the questionnaire method, we chose a quantitative research approach because the main focus was on exploring general differences between public and private experiences with technology in a systematic and experimental way. This has certain limitations, such as a self-report bias or a risk of misunderstanding items (e.g., Paulhus and Vazire, 2007; Remillard et al., 2014). In the future, field studies (e.g., in cafés or airports) are planned to complement findings from subjective self-reports with objective observations. Another methodological limitation concerns the fact that all analyses are based on recalled or imagined experiences, which yields the question of how representative these mental references are for real-life interactions. Though the here applied method to assess (retrospective) need fulfillment with a questionnaire or based on qualitative narratives is well established in HCI research (e.g., Hassenzahl et al., 2010, 2015; Partala and Kallinen, 2012; Partala and Kujala, 2016) and has proven as a valuable source of insights, future studies could complement retrospective approaches by a daily diary approach, such as experience sampling (Hektner et al., 2007). This *in-situ* method aims at measuring behavior, thoughts, and feelings of participants related to certain experiences or activities throughout their daily life and, thus, overcomes shortcomings of post-hoc techniques like recall errors. In doing so, this may deliver deeper insights into the source of positive affect, the purpose of interaction, and relevant context factors by instructing participants to report their daily experience with a specific kind of technology (e.g., virtual reality glasses) or technology interaction (e.g., voice control) over a longer period of time. Moreover, a qualitative research approach allows for the personalization of instruments, which facilitates more detailed addressing of sample characteristics (e.g., Zhang and Rau, 2015; Bolaños et al., 2021).

A second limitation concerns the experimental manipulation of context for the within-subject comparisons; it can be challenging to imagine your own feelings and behavior in fictional scenarios. Occasionally, participants’ recalled experiences that were difficult to imagine in an opposite context. For example, one person reported taking a picture of their family with a camera. Although we excluded participants who stated being unable to imagine their technology interaction in the modified context. Future studies could address this limitation by prescribing concrete technology interactions to ensure that the interaction is applicable to private and public contexts or concentrate on the interaction styles (e.g., touch-, voice-, or gestures-controlled). However, since the type of technology may influence the experience, it is recommended to shift the focus for examination from context-specific requirements to a specific technology type in future studies.

Third, the dichotomous classification of contexts as public and private constitutes a challenge. Participants’ ratings of how observed they felt during their experiences varied remarkably (in private and public). Moreover, interacting with a specific kind of technology can even feel more or less public when being surrounded by others. For example, two participants who recalled using VR glasses in public assessed the publicness of their interaction completely differently; while one participant reported that they felt strongly watched using VR glasses in a museum, the other participant did not feel observed at all during interacting in a gaming center. Presumably, potential spectators do not necessarily lead to a stronger feeling of being watched. Additionally, ubiquitous technologies blur the boundaries between private and public (Reeves, 2011), a clear distinction of public and private context is becoming increasingly difficult (Hatuka and Toch, 2016). Our study provides a coarse comparison between public and private that should be investigated in more detail in the future. Since most of the recalled and imagined public interactions were with familiar others in the present study, future studies could rely on the subjectively perceived publicness of interaction or focus on specific components of the social context (e.g., place of interaction, relationship to present others, and their involvement). It is presumed that the user experience of a given technology (interaction) may differ depending on whether present others are familiar or strangers (e.g., Williamson, 2012; Ahlström et al., 2014). Furthermore, since our original four-item publicness scale needed adjustments, we consider the development and validation of a scale to measure perceived subjective publicness of interactions an important topic.

Fourth, while our study on positive technology experience was limited to the experiences of users, future research should also involve the spectator’s perspective. More specifically, differences between users’ and spectators’ experiences of the very same technology interaction provide an interesting research subject. Previous studies that surveyed both, users and spectators, suggest that variations or deviations in acceptance ratings of technology between the two groups are likely (e.g., Lucero and Vetek, 2014; Koelle et al., 2015; Alallah et al., 2018). For example, while people enjoy using mobile phones in public or semi-public places (Hampton and Gupta, 2008), present others may be disturbed by forced-noticing of the user’s conversation (Campbell, 2007a,b). This public-private paradox (Jarvenpaa et al., 2005) should be further explored in experimental studies to deduct practical implications on how to design technology interactions that support users’ need fulfillment without negatively affecting present others – or even fulfilling their needs, too. Moreover, it could be interesting to investigate user-spectator dyads in further studies to explore potential differences in the technology experience of users and spectators depending on the extent of the spectator involvement in the technology interaction (e.g., Maurer et al., 2014; Gugenheimer et al., 2017; Humphreys and Hardeman, 2020). Such studies would contribute to a better understanding of socio-technical ecosystems by revealing, analyzing, and predicting interactions between user, spectator, and technology. Specifically, future studies could investigate how different forms of interaction (e.g., control *via* voice vs. touch) for a particular technology affect the social environment or present others, respectively, and how present peoples’ reactions, in turn, affect the user’s interaction process. Varying the role (i.e., active vs. passive involvement into user interaction) of the present others could provide further interesting insights.

Finally, the target sample is a German-speaking population; thus, generalizations to other cultural backgrounds have to be made with caution. Social contexts vary greatly within and across cultures. Comparative studies in different cultural contexts are needed to develop a more comprehensive picture (Sturm et al., 2015).

## Conclusion

Nowadays, many interactive products are still insensitive to our social surroundings or do not account for (sudden) contextual changes and, thus, are insufficiently adaptive to the socio-technical ecosystems we live in. As a consequence, these systems might be impaired in serving their intended purpose. Our work underlines the importance of adaptive technologies by showing that need fulfillment is dependent on social context and that people experience the same technology interaction less positive if this context is altered. In fact, our study is congruent with the findings of Hassenzahl et al. (2010, 2015) who found relatedness and popularity to be fulfilled to a greater extent in public contexts. However, we could not find support for the expected higher fulfillment of competence, security, and autonomy in private contexts, i.e., when being alone. Furthermore, need fulfillment of relatedness and popularity as well as positive affect significantly declined when originally present others were imagined absent. Thus, we conclude that positive experiences in public are mainly shaped by feelings of relatedness and popularity and are closely tied to the presence of other people. In addition to that, exploratory findings indicate that less negative affect is associated with higher social acceptability of the interaction, while positive affect is related to the overall fulfillment of psychological needs. Taken together, the technology experience differs systematically depending on the social context, which emphasizes the importance of considering contextual aspects in the design of interactive technologies. For example, design approaches for social contexts should consider ways to establish relatedness to present others and foster a popular impression of the user among them. While many research and design approaches focus on user and machine in a contextual vacuum, the dissemination of interactive products into everyday life calls for a more socially oriented perspective to create positive user experiences.

We hope that our work will stimulate further investigation into the role of social context for technology-mediated positive experiences. Existing models to describe and explain technology-mediated positive experiences focus mainly on the individual experience of the interactant. Neither do these models incorporate the impact of social context on individual experience, nor do they attempt to describe social context in detail. The present study demonstrates the impact of social context on individual experiences and is thus a first step toward the development of an expanded model that describes how social context shapes the relevance of different needs, their fulfillment, and ultimately subjective wellbeing. Therefore, future research should take a more fine-grained perspective on interaction in social context, for example, by including the spectator perspective, exploring dyad-interaction, and investigating further qualitative differences between different types of social contexts (see Uhde and Hassenzahl, 2021). In addition, further research methods, such as diary studies, should complement the present approach taken. At this point, our study already contributed to the understanding of the emergence of positive technology experiences in public situations in several regards. First and foremost, we experimentally manipulated the presence of others and thereby confirmed the existence of needs, which are paramount to public contexts. Second, we assessed a broad spectrum of psychological needs and could thus draw a more complete picture of public (as well as private) technology-mediated experiences. Finally, we provided evidence for a conceptual distinction between a rather preventative social acceptability view on technology use and a positive, need-based perspective on designing meaningful public technology-mediated experiences.

## Data Availability Statement

The datasets presented in this study can be found in online repositories. The names of the repository/repositories and accession number(s) can be found at: https://osf.io/s45v8/?view_only=9d4dbb6694094fd8a13db4dbfc9d25a2.

## Ethics Statement

Ethical review and approval were not required for the study on human participants in accordance with the local legislation and institutional requirements. The patients/participants provided their written informed consent to participate in this study.

## Author Contributions

PT, ST, and AU conceived and carried out the study and performed the data analyses. PT wrote the first draft of the manuscript. ST and AU revised the manuscript. SD and MH edited the drafts and provided feedback on the project and the manuscript. All authors contributed to the article and approved the submitted version.

## Conflict of Interest

The authors declare that the research was conducted in the absence of any commercial or financial relationships that could be construed as a potential conflict of interest.

## Publisher’s Note

All claims expressed in this article are solely those of the authors and do not necessarily represent those of their affiliated organizations, or those of the publisher, the editors and the reviewers. Any product that may be evaluated in this article, or claim that may be made by its manufacturer, is not guaranteed or endorsed by the publisher.
