# Supplementary material for: Technology-Mediated Experiences and Social Context: Relevant Needs in Private Vs. Public Interaction and the Importance of Others for Positive Affect
Source: Front Psychol. 2021 Sep 1;12:718315. doi: 10.3389/fpsyg.2021.718315 (PMC8440849; doi:10.3389/fpsyg.2021.718315)
Supplement: Supplementary file 1 [file Data_Sheet_1.PDF]

Supplementary Material

Table 1. Example report for all fourteen categories of interaction functions.

Function Category	Example Report
Entertainment	I installed an app on my smartphone to “replay” Harry Potter. I started the game, created a character, and was right back in the Harry Potter world. This world fascinated me as a child and teenager, and I loved to immerse myself into it.
Assistance	Since I was cold, I turned on the seat heater to increase the temperature on my side of the car via voice control. This was supported by a red LED light in parallel.
Household	I cooked with a Thermomix. In doing so, I managed to make a very tasty, healthy and quick dish that my family enjoyed very much.
Mobility	My mother was visiting Munich and we walked from my apartment towards the city center. On the way back, we would have had to wait longer for the subway, so it was faster to take an e-scooter. My mother had never used an e-scooter before and had trouble getting going at the beginning. We had quite a few laughs on the way home, especially when riding over cobblestones or lowered curbs.
Installation	I had ordered a new laptop online a few weeks earlier and was full of anticipation. When it arrived, I unpacked it right away and started setting it up. I transferred data and tried out new features. I was satisfied as my expectations were even exceeded.
Photography	I have had my first cell phone for a few months now. I discovered the selfie function for myself and was very happy about my first pretty photo of myself.
Relationship management	When my brother's wife gave birth to her first child, they still lived far away from us, so we couldn't visit spontaneously. That's why my parents and I then skyped with them and were able to have a look at my niece, which felt very emotional and beautiful.

Information gain	An interactive monitor is installed in front of the Tübingen City Museum. It can be used at any time (i.e., independent of the museum's opening hours). The monitor shows a city map with some color-separated hints/buttons pointing out a certain street, a certain building, etc. For example, if you select a building and click on it, a window appears with further choices from which you can again select and gain further information, e.g., from the categories historical or Tübingen at the time of National Socialism.
Navigation	I was standing at the subway station with friends and we all couldn’t remember how to get to a sushi restaurant. I opened Google Maps on my phone and entered the name of the restaurant. Then I used the route planner while walking and we followed the map on the phone. I put the phone back in my pocket as soon as I saw the restaurant.
Making music	A real piano is too impractical and too expensive for me. But digital pianos usually don't feel that real. Something is missing, but it is difficult to specify what. One day, I bought a Kawai Ca-95 digital piano, which has a so-called soundboard. It's hard to describe, but the first moment when I played it made me very happy. The whole case vibrated with every keystroke and I could not only hear but also feel every note, similar to a real grand piano. I improvised and, at that moment, was simply in symbiosis with this basically artificial object that does nothing but playing audio files with each keystroke. That feeling is still reproducible today: after all these years, when I sit down at my Kawai, they are still special moments for me. I often press a single key and rejoice in the great sound, the deep bass, and the physical resonance in the cabinet of the instrument.
Self-service	I used the automated checkout machine at the supermarket instead of standing in line at the regular checkout. Since I've done this many times before, I'm familiar with the process. I know which input fields to press, how to scan the goods, pay, and finally take out my receipt and hold it in front of a light barrier at the exit. This is faster than standing in the check-out line and feels especially good when other people eye the machines suspiciously or have to fight with them while I'm already able to leave the market.
Relaxation	The electronic heating blanket can be used before or during sleep and should warm the bed. Personally, I am very sensitive to cold, especially in winter, and often also freeze under a thick-down comforter. Therefore, it is every time a positive experience to be able to sleep in a pre-warmed bed. This experience is comparable to the experience of getting into a hot bath.

Cooperation	In online classes, I was able to set up working groups and then regretted each group separately. The motivation was exceptionally high due to the direct interaction, much better than in a single large group with 24 participants. The participants quickly mastered exchanging ideas among themselves and working on documents together. The group results were presented in the plenum. The team app was particularly good at supporting as I could be called into the groups if necessary.
Other	On a job hunt, I applied for an iOS developer position for a lark. Actually, I studied psychology and had only programmed as a hobby so far. After I was invited to the interview, I received a coding challenge. I had until the following day to implement several tasks. Since I actually managed to solve all the tasks - it was a positive experience to see the app working on my phone.

Table 2. Example report for all thirteen categories of interactive products.

Product Category	Example Report
Smartphone/Tablet	Recently, Spotify Kids got released in the App Store. I installed it on my daughter's tablet. It worked well and my daughter was very happy. It's great for kids and of course much better than the normal Spotify. My husband was also happy because now our Spotify list is no longer so “stuffed” with songs for kids.
Household appliance	I got myself a new smoothie blender and tried it out right away. Everything worked well and the smoothie was very tasty.
Vehicle	I rented a scooter for the first time and drove around with it. It was super nice weather, I felt really free and wished for this moment to never end.
Computer/Laptop	I was watching a movie from DisneyPlus on my computer (with a large extra screen).
Assistance system	While learning, I didn't know what a particular medical term meant and asked Alexa. Alexa was able to answer that efficiently right away. This made me happy because I was able to gain knowledge quickly and without much effort.

Game console	I played a video game together with my parents and my partner. It was a digital board game (Wii Party) which we played on my mother's Wii in my parents' living room. Playing together, was a lot of fun for all of us, especially because of the motion control of the Wii.
Virtual reality	I used VR glasses in a gaming center. You saw yourself in a laser tag arena and could "shoot" other VR figures. The sound and visuals were pretty realistic.
Camera	I used my Canon camera at my nephew's school enrollment party to take a picture of the whole family.
Wearable	I tested my new Garmin smartwatch. It allows me to exercise without a cell phone, track my activities as accurately as possible, and use my Spotify playlists with Bluetooth headphones, i.e., without annoying cables.
Audio system	In the evenings, I like to lie on the sofa to relax and listen to audio books on my stereo. I have a particularly positive memory of an evening when I, at the same time, was doing a puzzle and drinking hot chocolate.
Television	I watched the movie Paddington 2 with a friend in his parents' living room.
Musical instrument	I was repairing my friend's electric piano. My father had soldered for me one of the circuit boards where one solder joint had been dead. I reinstalled the circuit board, which required taking the whole piano apart and put it back together. That was fun and I felt like an electrical professional.
Other	I interacted with a robot during a seminar. It answered appropriately and also helped me.
